# A Trajectory Privacy Preserving Scheme in the CANNQ Service for IoT

**DOI:** 10.3390/s19092190

**Published:** 2019-05-12

**Authors:** Lin Zhang, Chao Jin, Hai-ping Huang, Xiong Fu, Ru-chuan Wang

**Affiliations:** 1 School of Computer, Nanjing University of Posts and Telecommunications, Nanjing 210003, China; 15195886318@163.com (C.J.); hhp@njupt.edu.cn (H.-p.H.); fux@njupt.edu.cn (X.F.); wangrc@njupt.edu.cn (R.-c.W.); 2 Jiangsu High Technology Research Key Laboratory for Wireless Sensor Networks, Nanjing 210003, China; 3 Institute of Computer Technology, Nanjing University of Posts and Telecommunications, Nanjing 210003, China

**Keywords:** aggregate nearest neighbor query, trajectory privacy, spatial K-anonymity, secure areas, strategy optimization

## Abstract

Nowadays, anyone carrying a mobile device can enjoy the various location-based services provided by the Internet of Things (IoT). ‘Aggregate nearest neighbor query’ is a new type of location-based query which asks the question, ‘what is the best location for a given group of people to gather?’ There are numerous, promising applications for this type of query, but it needs to be done in a secure and private way. Therefore, a trajectory privacy-preserving scheme, based on a trusted anonymous server (TAS) is proposed. Specifically, in the snapshot queries, the TAS generates a group request that satisfies the spatial K-anonymity for the group of users—to prevent the location-based service provider (LSP) from an inference attack—and in continuous queries, the TAS determines whether the group request needs to be resent by detecting whether the users will leave their secure areas, so as to reduce the probability that the LSP reconstructs the users’ real trajectories. Furthermore, an aggregate nearest neighbor query algorithm based on strategy optimization, is adopted, to minimize the overhead of the LSP. The response speed of the results is improved by narrowing the search scope of the points of interest (POIs) and speeding up the prune of the non-nearest neighbors. The security analysis and simulation results demonstrated that our proposed scheme could protect the users’ location and trajectory privacy, and the response speed and communication overhead of the service, were superior to other peer algorithms, both in the snapshot and continuous queries.

## 1. Introduction

In recent years, with the popularization of mobile portable devices, advancement in spatial positioning technology and development of Internet of Things (IoT), location-based services (LBS) have successfully appeared in public view. Various personalized services, combined with location elements, such as location sharing [1,2], nearest neighbor query [3,4], friend discovery [5,6], etc., are popular among users. Among them, the k nearest neighbor (kNN) queries can find the k points of interest (POIs) nearest to the users, e.g., find three gas stations nearest to me. However, with the emergence of new scenarios, the kNN queries have been unable to meet more complex needs of users. For example, if several friends in different places are going to have dinner after work, how do you find a western restaurant nearest to them? If several users in different locations plan to carpool, how do you help the driver to plan the best route to pick up all passengers? Tan [7] defines these scenarios as ‘multiple object convergence’, and the corresponding query mode is called ‘aggregate nearest neighbor (ANN) query’. In the ANN queries, multiple users (at least two) need to send query requests to the location-based service provider (LSP) by building query groups. The list of nearest neighbors returned by the LSP, depends on the specified aggregate functions, which includes ‘sum’, ‘max’, and ‘min’. Different aggregate functions cause differences in query results, where the ‘sum’ function makes sure that the total distance traveled by all persons is minimum, while the ‘max/min’ function aims to minimize the maximum/minimum traveling distance requested by any person. This paper focuses on the ‘sum’ function because it properly takes into account the distance traveled by everyone in the group.

**Figure 1:**
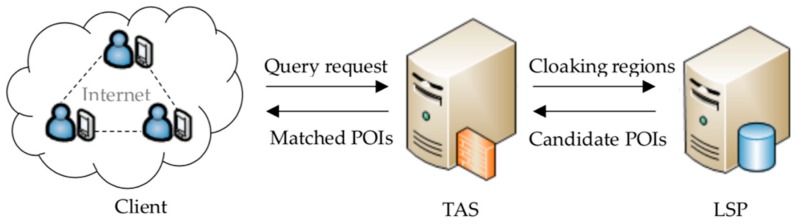
The architecture based on a trusted anonymous server.

However, a necessary condition for the widespread use of IoT is to protect the privacy of users, especially location privacy. As we all know, the LSP requires the inquirers to provide their location information to obtain the service. The more accurate the location that the user sends, the higher quality of the service the user receives, but the more privacy the user leaks [8]. Therefore, prevention of the LSP from obtaining the user’s location information during the process of obtaining the service is an urgent problem to be solved. For example, in academia, most [9,10,11,12] adopt an architecture, based on a trusted anonymous server (TAS). As shown in Figure 1, the TAS can help users to send query requests, which avoids direct communication between users and the LSP. In addition, some scholars have questioned that the TAS will become a performance bottleneck because all query requests need to pass through it, thus, increasing the response delay of the overall service. Therefore, balancing privacy protection and service response delay is another urgent problem to be solved.

For current surveys on location privacy protection in the IoT, there is not yet much research available on ‘the continuous aggregate nearest neighbor query service that protects the location and trajectory privacy of the group of users’ (PCANNQ). The main problems that exist are listed below.

First, in the snapshot queries, it is feasible to introduce the TAS into PCANNQ, to protect the user’s location privacy, but there is a little adjustment for protecting the location privacy of the group of users.

Second, in continuous queries, it is feasible to protect the user’s trajectory privacy by reducing the communication frequency between the user and the LSP, but there is a lack of a query scheme that protects the trajectory privacy of the group of users, while ensuring accurate query results.

Third, it is feasible to improve the response speed of the queries by improving the ANN query algorithm, but there is a lack of an efficient method for computing the aggregate nearest neighbors.

The rest of this paper is organized as follows. Section 2 presents the related work; Section 3 provides a general overview of our system model; Section 4 elaborates details of our proposed scheme and analyzes the security; Section 5 discusses the construction of circular secure areas; and Section 6 discusses the ANN query algorithm based on strategy optimization. Extensive simulation experiments were carried out and our findings have been reported in Section 7. Finally, Section 8 concludes the paper with some directions for future work.

## 2. Related Work

In this section, we reviewed certain available research on the main techniques in the IoT, including private nearest neighbor query schemes, and a new type of query—aggregate nearest neighbor query.

### 2.1. Private Nearest Neighbor Query Scheme

Private nearest neighbor queries include snapshot queries that protect location privacy [13,14,15,16] and continuous queries that protect trajectory privacy [17,18,19,20].

In snapshot queries, location privacy protection techniques mainly included location perturbation [13], spatial cloaking [14,15], and spatial transformation [16]. In Reference [13], the Space Twist technique sent a false location information to the LSP, to query the nearest neighbors about the fake location. The method was simple and did not require the intervention of any third party, but the communication efficiency was very low, because the number of single-inquiry communication was indefinite, which made it difficult to respond to continuous queries in a timely and stable manner. The Casper technique proposed in Reference [14] involved the location of an anonymous server and a private query processor. The method showed a high efficiency for the nearest neighbor queries on the cloaking regions. The basic idea of the spatial cloaking technique was to blur a user’s exact location into a cloaked area that satisfied the user specified privacy requirements. Later, Chow [15] proposed a spatial cloaking algorithm for mobile P2P environments. In Reference [16], the Hilbert curve encryption technology was adopted to achieve privacy protection, but the query results were not completely accurate.

In continuous queries, trajectory privacy protection techniques mainly included reducing the number of data submissions [17,18] and submitting redundant queries [19,20]. Zhang [17] proposed a caching-based scheme to enhance user privacy which employed a two-level caching to cache result data. However, this scheme did not consider the user’s neighbor cache and the anonymity, based on user mobility. To further enhance user privacy and increase the cache hit rate, Zhang [18] adopted a multi-level caching to cache users’ results. In fact, this paper focused on using caching technology to cache the query results for users’ future queries. When other users issued similar queries in the future, the results could be retrieved from the cache of the TAS without having to submit a request to the LSP, thereby, protecting the privacy of the users. Hwang [19] required the TAS issued redundant queries with K-anonymizing spatial regions (K-ASRs) and randomized the sequence of the query time, in order to protect the trajectory privacy. Inspired by Reference [19], Peng [20] proposed a collaborative trajectory privacy preserving (CTPP) scheme, which constructed the K-ASRs based on the gathered information obtained from multi-hop peers and the trajectory privacy was guaranteed by user collaboration. This method broke the correlations of the continuous LBS queries to obfuscate the user’s actual trajectory.

### 2.2. Aggregate Nearest Neighbor Query

Aggregate nearest neighbor (ANN) query [21,22,23,24,25,26,27] is a variant of the kNN query used to help multiple users to find the optimal meeting place. Papadias [21] first proposed the concept of ANN query, and three proposed algorithms, including multiple query method (MQM), single point method (SPM), and minimum bounding method (MBM) suitable for Euclidean space, but the query efficiency was low due to the large search scope. At the same time, Yiu [22] applied the ANN query to the road networks. In order to optimize queries and improve query efficiency, Luo [23] proposed a Vp-ANN query algorithm, based on a ‘two-points projection’. The algorithm, first, projects the query points to specific rows, analyzes their distribution, and then trims the search scope. The algorithm reduces the search space to some extent. The voronoi-based ANN query algorithm proposed by Sun [26] divides the query algorithm into the search phase and the prune phase. The search phase computes the nearest neighbors of each query point in some order, to obtain the candidate POIs, until all the query points find a common POI. The prune phase filters out the unqualified POIs, according to the prune strategy of a given aggregate function, until only one candidate POI remains as the final result. This algorithm is currently the most reasonable, but the search strategy and prune strategy are not optimal, and there is still room for improvement.

Additionally, the index structure of POIs is also an important factor that affects the query efficiency. Sun [23]’s use of R tree [28] to organize POIs has actually proved to be inefficient. Although there have been more efficient indexing techniques to help queries, for example, the VOR tree [29], the AVR tree [30], etc., which satisfies a faster search speed, but the purpose of this paper is to improve the search and prune strategy, and highlight the superiority of the proposed strategy. Therefore, the R tree spatial index is still used to achieve comparability between the query algorithms.

### 2.3. Our Contributions

Location-based query services are the basic functions of the IOT, and the leakage of location and trajectory privacy is a widespread problem. This paper aims to design a secure and efficient PCANNQ scheme, to protect the location and trajectory privacy of multiple users, while ensuring the overall performance of the service. In other words, our scheme emphasizes the continuous and rapid response of this type of queries, while protecting the location and trajectory privacy of the group of users. Therefore, there is a fundamental difference from the above-related researches. Our contributions in this paper can be summarized as follows.

(1) We propose a new PCANNQ scheme based on the TAS. The TAS helps the group of users to send group requests that satisfy spatial K-anonymity to the LSP. It prevents the LSP from directly obtaining the real location of the group of users, thus, protecting the location privacy of the group of users in snapshot queries.
(2) We propose a circular secure areas construction method suitable for continuous queries. On one hand, the method ensures that the optimal aggregate nearest neighbor remains unchanged when the users move within the areas, on the other hand, the secure areas can conceal the real locations of the users, thus, reducing the probability of the LSP reconstructing the users’ trajectories.
(3) We propose an efficient aggregate nearest neighbor query algorithm. The algorithm includes a search phase and a prune phase. The search phase is designed to limit the minimum search scope of POIs, based on the aggregate subgroup, and the prune phase is designed to filter the non-nearest neighbors quickly, thus, improving the response speed of the query algorithm.
(4) We experimentally compared the performance of our scheme with peer algorithms, in terms of security, average processing overhead, query efficiency, etc. The experimental results showed that our scheme could protect the location and trajectory privacy of the group of users, while ensuring the overall performance of the service, which achieved the expectation of the paper.

## 3. System Model and Definition

This section first analyzes the privacy leaks in the location-based query services. Then, the main entities involved in the system are introduced. Finally, we defined continuous aggregate nearest neighbor queries.

### 3.1. Problem Statement

We consider the following scenario. Alice, Bob, and Carl plan to have dinner after work and query some western restaurants nearest to them. Due to the rush hour, the road conditions of each user are unknown, and traffic jams might occur. Therefore, users need the LSP to update more appropriate places in real time, during their movement, so that they can meet more quickly.

**Figure 2:**
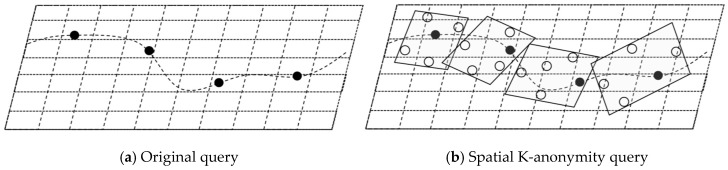
Original query and K-anonymity query of single user.

There are two concerns—first, in snapshot LBS queries, users need to send their current locations to the untrusted LSP, to obtain the service; second, in the continuous LBS queries, users need to send their current locations to the untrusted LSP, within a fixed time interval. This increases the risk of exposure of users’ location and trajectory privacy. Figure 2a shows the service usage of a user in the LBS queries. The LSP can construct the user’s mobile trajectory, by collecting the user’s location sequence, and then infer the user’s private information, such as interests, habits, and religions.

For the first issue, the spatial K-anonymity method can be used, which generates a cloaking-region-satisfied K-anonymity, every time the user issues a query. The region contains k-1 dummy locations and a real location, which are indistinguishable. However, for the second issue, there is a risk of failure. Take Figure 2b as an example, the method generates cloaking-regions-satisfied 5-anonymity for a user, at each timestamp, in continuous queries. On the one hand, there is a spatial correlation between locations. By connecting the cloaking regions, the spatial characteristics of the original trajectory, such as the moving path and direction, can still be inferred. On the other hand, there is a temporal correlation between the locations. The LSP can reconstruct the trajectory of the user with great probability, according to the sequence of the query issuing time. We can see that in this scenario, although each snapshot query is protected, based on the K-anonymity principle, the trajectory of the user can still be easily disclosed.

### 3.2. System Model

To address the issues mentioned above, we propose a PCANNQ scheme for the IOT. The architecture is shown in Figure 1 and details will be explained in Section 4. It consists of three main entities: Clients, TAS, and LSP. Their main functions can be described as follows:

(1) **Client.** Each mobile client carries a mobile device with functions, such as information processing, data storage, and global positioning (e.g., GPS) to quickly connect to the IOT. The users participating in the LBS queries trust each other, and there is no collusion attack between any users and the LSP.
(2) **TAS.** The TAS is trusted. Its main functions include concealing users’ identities, generating cloaking regions satisfying the spatial K-anonymity for users, and filtering out the query results belonging to the real user, in the query results. In the continuous queries, TAS can also generate secure areas, according to whether the query result changes, which dynamically conceal the users’ moving paths.
(3) **LSP.** The LSP is semi-trusted. On one hand, the LSP stores map resources and location-related POIs, such as restaurants, hospitals, and tourist attractions, to provide users with timely location-based query services. On the other hand, users do not know whether the LSP-stored trajectory data generated in the query process, and whether the private information contained in the trajectories is abused or not, is also unknown.

Therefore, the LSP is considered as an adversary in this paper. For the location information collected in the snapshot queries, the LSP attempts to determine the location related to the user’s true identity, and then infers other private information, based on the location. For example, Alice often queries nearby drivers at around 17:00. It could be inferred that the location might be her workplace.

For the trajectory data collected in continuous queries, the LSP could obtain more information about the user by analyzing the trajectory data. For example, Alice goes to work at 08:00, gets off work at 17:00, and often goes to Starbucks at noon. Therefore, the goal of the LSP is to capture as many of the users’ continuous locations as possible, and then reconstruct the users’ complete trajectories.

### 3.3. Continuous Aggregate Nearest Neighbor Query

Continuous aggregate nearest neighbor queries (CANNQ) refer to a group of users querying the latest aggregate nearest neighbors, during their movement, which is usually represented by a quadruple called CANNQ=<Q,P,T,NN>.

$Q=\left\{q_{1},q_{2},\cdots ,q_{n}\right\}$ represents the group of users that issued the queries where $q_{i}=<{\mathrm{Id}}_{i},x_{i},y_{i},t_{i}>$. ${\mathrm{Id}}_{i}$, $x_{i}$ and $y_{i}$ represent $q_{i}’$s identity, latitude, and longitude, respectively.

$P=\left\{p_{1},p_{2},\cdots ,p_{m}\right\}$ represents the collection of POIs where $p_{i}=<x_{i},y_{i},{\mathrm{type}}_{i}>$. $x_{i}$, $y_{i}$ and ${\mathrm{type}}_{i}$ represent $p_{i}’s$ latitude, longitude, and type, respectively.

$T=\left\{t_{1},t_{2},\cdots ,t_{o}\right\}$ represents the time sequence of the group of users from issuing the query to ending the query where $t_{1}$ and $t_{o}$ represent start and end times, respectively.

${\mathrm{NN}}_{t\in T}=\{p|{\mathrm{dist}}_{\mathrm{agg}}\left(p_{i},Q\right)\leq {\mathrm{dist}}_{\mathrm{agg}}\left(p_{j},Q\right),i,j\in \left[1,n\right],i\neq j\}$ represents a list of the nearest neighbors of the group of users at time t, where the POIs are sorted in ascending order, by ${\mathrm{dist}}_{\mathrm{agg}}\left(p,Q\right)$.

${\mathrm{dist}}_{\mathrm{agg}}\left(p,Q\right)=\sum_{k\in \left[1,n\right]}\mathrm{dist}\left(p,q_{k}\right)$ represents the total distance from p to all users in Q.

**Figure 3:**
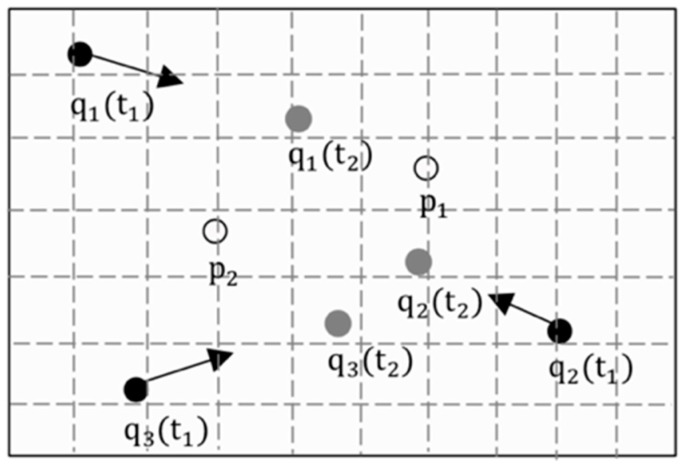
Example of a continuous aggregate nearest neighbor queries (CANNQ).

Figure 3 shows an example of CANNQ. By the definition of CANNQ, $Q=\left\{q_{1},q_{2},q_{3}\right\}$, $P=\left\{p_{1},p_{2}\right\}$, $T=\left\{t_{1},t_{2}\right\}$. At time $t_{1}$, the list of nearest neighbors ${\mathrm{NN}}_{t_{1}}=\left\{p_{2},p_{1}\right\}$ where $p_{2}$ is the aggregate nearest neighbor, and at time $t_{2}$, the list of nearest neighbors ${\mathrm{NN}}_{t_{2}}=\left\{p_{1},p_{2}\right\}$, where $p_{1}$ is the aggregate nearest neighbor.

## 4. Our PCANNQ Scheme

In this section, first, we introduced the functions of entities in our system. Then, we elaborated the work process of the PCANNQ scheme. Finally, we analyzed the privacy security of the PCANNQ scheme. The important notations used in the PCANNQ scheme are listed in Table 1 for reference.

### 4.1. System Architecture

**Figure 4:**
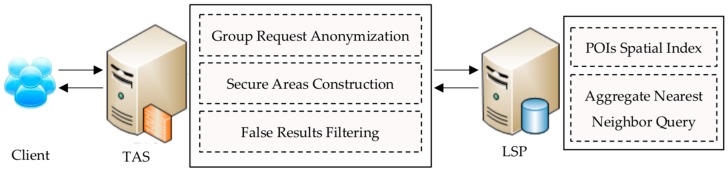
Details of our system architecture.

To enhance the privacy of users, we proposed a new PCANNQ scheme. Figure 4 shows the system architecture and some details. The TAS is the core component of this architecture and is responsible for privacy protection. The main functions include—group request anonymization, secure areas construction, and false results filtering. The LSP is a location service provider. The main functions include—POIs data loading and aggregate nearest neighbor query.

Although adding the TAS between users and the LSP will inevitably add an extra privacy protection overhead, it is reasonable, because (1) the cost of privacy protection accounts for a small proportion of the total service cost. (2) Users do not need to maintain real-time communication with the LSP, because the query results at some moments can be directly obtained from the cache of the TAS, so the efficiency of the overall service is improved, and users leak fewer locations to the LSP. (3) The LSP only needs to maintain the POIs data and the query algorithm itself, and then throw the results to the TAS, so the function is simpler.

### 4.2. Process of PCANNQ

The work process of PCANNQ is shown as follows.

**Figure 5:**
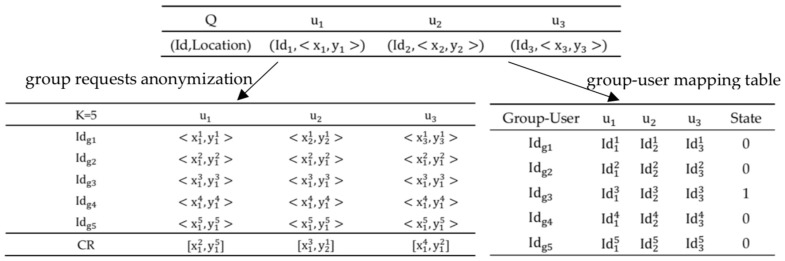
Anonymous process.

Step1. Users Q send requests to the TAS to build a query group. ${\mathrm{req}}_{q\in Q}=\left(q,P.\mathrm{type}\right)=\left({\mathrm{Id}}_{q},<x_{q},y_{q}>,P.\mathrm{type}\right)$ where ${\mathrm{Id}}_{q}$, $<x_{q},y_{q}>$ and P.type represents the identity of user q, the location of user q and the type of POIs, respectively.
Step2. After receiving the request ${\mathrm{req}}_{q}$, the TAS determines whether it is the first time for the user group to issue the query, if so, turn to Step 3–7, and if not, turn to Step 8.
Step3. The TAS constructs the cloaking regions for every user in the group, which covers their true locations to satisfy spatial k-anonymity, and then converts these cloaking regions into k group requests sent to the LSP. ${\mathrm{req}}_{i\in \left[1,k\right]}=\left\{{\mathrm{Id}}_{g_{i}},<x_{1}^{i},y_{1}^{i}>,<x_{2}^{i},y_{2}^{i}>,\cdots ,<x_{n}^{i},y_{n}^{i}>,P_{i}.\mathrm{type}\right\}$ where ${\mathrm{Id}}_{g}$ represents the identity of the group which replaces ${\mathrm{Id}}_{q}$. As shown in Figure 5, the TAS represents all users’ Id as a group Id, and five indistinguishable group requests are generated in this anonymous process.
Step4. The TAS maintains a group-user mapping table locally, which records the corresponding relationship between the group Id and user Id. When the LSP returns the results, the table can help the TAS to quickly filter out the false results. As shown in Figure 5, the number of users |Q|=3, the degree of anonymity k=5. A record with a value of 1 in the ‘State’ field (e.g., the third record) represents the real group of users, and the other items are k-anonymized false records.
Step5. The LSP performs an ANNQ, based on strategy optimization (see Section 6 for details) for all requests, generates a lists of nearest neighbors and then returns the results to the TAS. ${\mathrm{res}}_{i\in \left[1,k\right]}=\left\{{\mathrm{Id}}_{g_{i}},\mathrm{NN}\right\}$ where $\mathrm{NN}=\left\{p_{\mathrm{ann}}^{1},p_{\mathrm{ann}}^{2},\ldots ,p_{\mathrm{ann}}^{m}\right\}$.
Step6. The TAS filters the results based on the group-user mapping table and returns the true neighbor lists to the true group of users.
Step7. The TAS calculates the radius by $\mathrm{dist}{\left(p_{\mathrm{ann}}^{1},Q\right)}_{\max}$ and $\mathrm{dist}{\left(p_{\mathrm{ann}}^{2},Q\right)}_{\max}$, and constructs circular secure areas for the group of users (see Section 5 for details).
Step8. The TAS monitors the group of users in real-time to see whether the users exceed their secure areas. If someone exceeds, the user group is prompted to update the query results. If the request needs to be resubmitted, turn to Step 3. If not, continue to monitor until the group of users end the query.

### 4.3. Security Analysis

**Figure 6:**
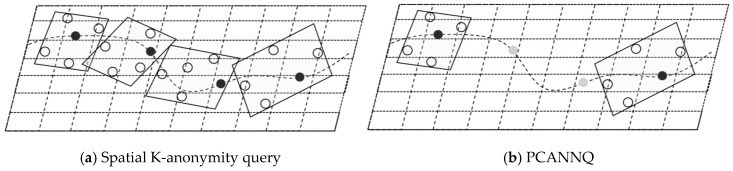
K-anonymity query and PCANNQ.

First, we analyzed the location security in the snapshot queries and the ability of our scheme to resist the inference attack of the LSP. As shown in Figure 6a, the degree of anonymity K = 5. At each timestamp, the TAS randomly generated 4 dummy locations to conceal the real location. Since the LSP had background knowledge of the query probability of each location, the LSP could easily filter out some dummy locations (e.g., mountains, lakes) when the query probabilities of the randomly generated dummy locations were different, so the spatial K-anonymity method could not resist the inference attack of the LSP. Therefore, an entropy-based spatial K-anonymity method was adopted, where entropy represented the uncertainty of determining the true location from the candidate set, which was used to measure the anonymity of the method. Specifically, the entropy could be represented by H, as shown in Formula (1).

(1) $$H=-\sum_{i=1}^{k}p_{i}\cdot \log_{2}p_{i}$$

The maximum entropy $H_{\max}=\log_{2}k$ was reached when the k locations had the same probability 1/k. Therefore, our method selected the locations with the same query probability as the dummy locations, to resist the inference attack of the LSP.

Then, we analyzed the trajectory security in continuous queries and the ability of our scheme to resist the spatial-temporal correlation attacks of the LSP. As mentioned in Figure 2b, the cloaking regions in Figure 6a showed a spatial-temporal correlation. When the LSP detected that all locations of a certain user were in these regions, it could infer that the user had a high probability of being the user who issued the query, and then reconstructed the user’s trajectory. Figure 6b shows the location submission in the PCANNQ scheme, where the black dots (e.g., $t_{1}$, $t_{4}$) were submitted to the LSP and the gray dots ($t_{2}$, $t_{3}$) were uncommitted because they were concealed in the secure area.

The principle of the PCANNQ scheme is that the TAS deconstructs continuous queries into irrelevant snapshot queries, and the secure areas can conceal part of the users’ trajectories, so that the cloaking regions generated in each query lose the spatial-temporal correlation. Therefore, even if the LSP monitors that a user is completely in these discrete cloaking regions, it cannot reconstruct their trajectory. We use η to measure the security of trajectory privacy, as shown in Formula (2).

(2) $$\eta =\frac{\#\mathrm{actual}\;\mathrm{queries}}{\#\mathrm{total}\;\mathrm{queries}}$$

Given the total number of queries, fewer actual queries means less private information is exposed to the LSP and a lower probability that the LSP reconstructs the user’s trajectory.

Theoretically, our PCANNQ scheme can effectively resist the inference attack and the spatial-temporal correlation attack of the LSP.

## 5. Circular Secure Areas

In this section, we first introduced the motivation and the function of secure areas. Then, we elaborated on the verification and construction method of secure areas. Finally, we designed a PCANNQ based on the circular secure areas.

### 5.1. Motivation

Conventional continuous queries were usually converted to snapshot queries at each timestamp. However, it was found that not every time the location of the user changed did it lead to a change of the optimal nearest neighbor. The reason was that the user had a secure area with respect to the current optimal nearest neighbor, that is to say, this area was dominated by the optimal nearest neighbor. When the user moved in the area, the optimal nearest neighbor was always the same. When the user left the area, the query result was changed to other nearest neighbors.

Similarly, in a PCANNQ, users in the group also had their own secure areas, with respect to the current optimal aggregate nearest neighbor. When users moved in their secure areas, the optimal aggregate nearest neighbor was always the same, so the group did not have to continue to send query requests to the LSP, which meant that the users reduced the exposure of their trajectories. Therefore, the purpose of this section was to calculate the secure areas for the group of users.

**Definition** **1.** **Secure Areas.** Given a group of users $Q=\left\{q_{i}|\begin{array}{c} n\\ i=1 \end{array}\right\}$ and a group of areas $R=\left\{R_{i}|\begin{array}{c} n\\ i=1 \end{array}\right\}$, if $\forall \langle q_{1},q_{2},\cdots ,q_{n}\rangle \in R_{1}\times R_{2}\times \cdots \times R_{n}$ which guarantees that the optimal aggregate nearest neighbor $p^{*}$ is invariable, then R are called secure areas.

**Figure 7:**
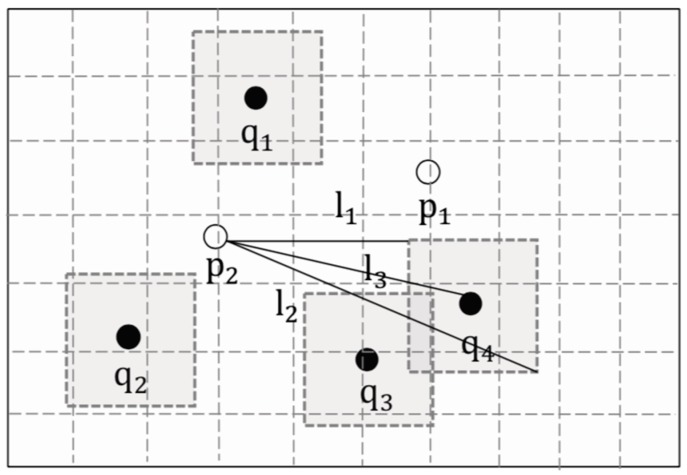
Secure areas and dominated distances.

As shown in Figure 7, we assumed that the dotted line was the moving boundary of each user. When users moved in their own areas, their optimal aggregate nearest neighbor was always $p_{1}$. When the boundary was crossed, the aggregate nearest neighbor changed to other points, such as $p_{2}$.

**Definition** **2.** **Maximum Secure Areas.** Given a group of users $Q=\left\{q_{i}|\begin{array}{c} n\\ i=1 \end{array}\right\}$ and a group of areas $R^{*}=\left\{R_{i}^{*}|\begin{array}{c} n\\ i=1 \end{array}\right\}$, if $\forall R^{\prime }=\left\{R_{i}^{\prime }|\begin{array}{c} n\\ i=1 \end{array}\right\}$, $R^{\prime }\neq R^{*}$, satisfy $R_{i}^{\prime }\subseteq R_{i}^{*}$, then $R^{*}$ are called maximum secure areas.

Of course, the larger the secure areas, the higher the privacy security, but it also brings greater computing overhead, so this study aimed to construct more efficient secure areas.

After this, we considered under what conditions the constructed areas formed the secure areas (see details in Section 5.1) and how could the secure areas be maximized (see details in Section 5.2).

### 5.2. Verification of Secure Areas

If $R=\left\{R_{i}|\begin{array}{c} n\\ i=1 \end{array}\right\}$ are secure areas, then the conditions of definition 1 should be satisfied, but there are infinite number of combinations of user locations in these areas. It is not feasible to test all instances one by one, but we can find the critical value from the first aggregate nearest neighbor to the second aggregate nearest neighbor. The verification method is as follows.

**Definition** **3.** **Dominated Distance.** *Given a group of users $Q=\left\{q_{i}|\begin{array}{c} n\\ i=1 \end{array}\right\}$ and p∈P, the dominated distance of p is ${\mathrm{dist}}_{\mathrm{dom}}\left(p,Q\right)$, as show in Formula (3).*

(3) $${\mathrm{dist}}_{\mathrm{dom}}\left(p,Q\right)=\underset{q\in Q}{\max}\left(\mathrm{dist}\left(p,q\right)\right)$$

**Definition** **4.** **Max/minimum Dominated Distance.** *Given p∈P and area set $R=\left\{R_{i}|\begin{array}{c} n\\ i=1 \end{array}\right\}$, the maximum dominated distance of p is ${\mathrm{dist}}_{\mathrm{dom}\_\mathrm{up}}\left(p,R\right)$, as shown in Formula (4).*

(4) $${\mathrm{dist}}_{\mathrm{dom}\_\mathrm{up}}\left(p,R\right)=\underset{R_{i}\in R}{\max}\left(\mathrm{dist}{\left(p,R_{i}\right)}_{\max}\right)$$

*the minimum dominated distance of p is ${\mathrm{dist}}_{\mathrm{dom}\_\mathrm{down}}\left(p,R\right)$, as shown in Formula (5).*

(5) $${\mathrm{dist}}_{\mathrm{dom}\_\mathrm{down}}\left(p,R\right)=\underset{R_{i}\in R}{\max}\left(\mathrm{dist}{\left(p,R_{i}\right)}_{\min}\right)$$

As shown in Figure 7, we suppose the grey areas are the secure areas. Take $p_{2}$ as an example, the dominated distance of $p_{2}$ is ${\mathrm{dist}}_{\mathrm{dom}}\left(p_{2},Q\right)=l_{3}$, the maximum dominated distance of $p_{2}$ is ${\mathrm{dist}}_{\mathrm{dom}\_\mathrm{up}}\left(p,R\right)={\;l}_{2}$, the minimum dominated distance of $p_{2}$ is ${\mathrm{dist}}_{\mathrm{dom}\_\mathrm{up}}\left(p,R\right)={\;l}_{1}$.

If the set of secure areas $R=\left\{R_{i}|\begin{array}{c} n\\ i=1 \end{array}\right\}$ belongs to the current optimal aggregate nearest neighbor, that is to say, when the users are in their respective secure areas, $p^{*}$ will not be replaced by any other $p\in P-\left\{p^{*}\right\}$, then the dominated distance of $p^{*}$ should be guaranteed to be less than or equal to that of p. The Theorem 1 is shown below.

**Theorem** **1.** Given a group of users $Q=\left\{q_{i}|\begin{array}{c} n\\ i=1 \end{array}\right\}$ and a set of secure areas $R=\left\{R_{i}|\begin{array}{c} n\\ i=1 \end{array}\right\}$, if $\forall p\in P-\left\{p^{*}\right\}$, satisfy

(6) $${\mathrm{dist}}_{\mathrm{dom}\_\mathrm{up}}\left(p^{*},R\right)\leq {\mathrm{dist}}_{\mathrm{dom}\_\mathrm{down}}\left(p,R\right)$$

then ${\mathrm{dist}}_{\mathrm{dom}}\left(p^{*},Q\right)\leq {\mathrm{dist}}_{\mathrm{dom}}\left(p,Q\right)$.

**Proof.** 

1. Let $Q_{\mathrm{test}}=\left\{q_{i}|\begin{array}{c} n\\ i=1 \end{array}\right\}\in R$.
2. By definition 4, ${\mathrm{dist}}_{\mathrm{dom}}\left(p^{*},Q_{\mathrm{test}}\right)\leq {\mathrm{dist}}_{\mathrm{dom}\_\mathrm{up}}\left(p^{*},R\right)$, ${\mathrm{dist}}_{\mathrm{dom}\_\mathrm{down}}\left(p^{*},R\right)\leq {\mathrm{dist}}_{\mathrm{dom}}\left(p,Q_{\mathrm{test}}\right)$.
3. $∵{\mathrm{dist}}_{{\mathrm{dom}}_{\mathrm{up}}}\left(p^{*},R\right)\leq {\mathrm{dist}}_{{\mathrm{dom}}_{\mathrm{down}}}\left(p,R\right)$.
4. $∴{\mathrm{dist}}_{\mathrm{dom}}\left(p^{*},Q\right)\leq {\mathrm{dist}}_{\mathrm{dom}}\left(p,Q\right).$

□

### 5.3. Construction of Secure Areas

**Figure 8:**
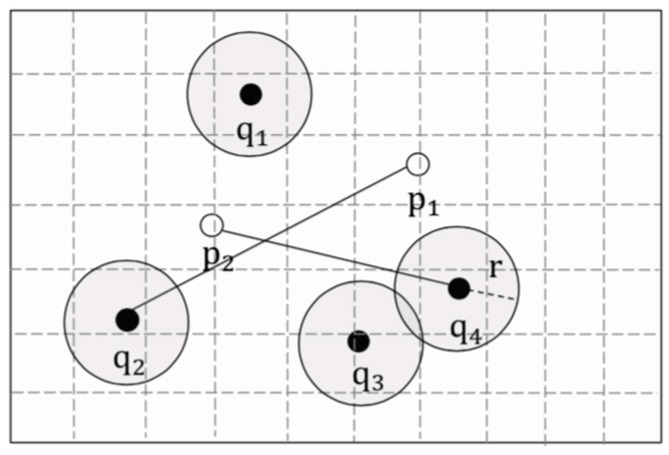
Circular secure areas.

Considering that the secure area should be constructed as conveniently as possible, this study approximated the secure area as a circle, as shown in Figure 8. At the same time, the radius of the circular area should be as large as possible, in order to reduce the communication frequency between the user and the LSP. Theorem 2 is used to calculate the maximum radius of a circular secure area, so that the areas remain valid for the current optimal aggregate nearest neighbor.

**Theorem** **2.** Given a group of users $Q=\left\{q_{i}|\begin{array}{c} n\\ i=1 \end{array}\right\}$ and a set of secure areas $R=\left\{R_{i}|\begin{array}{c} n\\ i=1 \end{array}\right\}$ where $R_{i}=C\left(q_{i},r_{\max}\right)$ represents a circle with center $q_{i}$ and radius $r_{\max}$. If the current optimal aggregate nearest neighbor $p_{\mathrm{ann}}^{1}=p^{*}$, then

(7) $$r_{\max}=\frac{\underset{p\in P-\left\{p^{*}\right\}}{\min}\left(\mathrm{dist}{\left(p,Q\right)}_{\max}\right)-\mathrm{dist}{\left(p^{*},Q\right)}_{\max}}{2}$$

**Proof.** 

1. Let $R_{i}=C\left(q_{i},r\right)$.
2. By definition 7, ${\mathrm{dist}}_{\mathrm{dom}\_\mathrm{up}}\left(p^{*},R\right)=\underset{R_{i}\in R}{\max}\left(\mathrm{dist}{\left(p^{*},R_{i}\right)}_{\max}\right)=\underset{q_{i}\in Q}{\max}\left(\mathrm{dist}\left(p^{*},q_{i}\right)+r\right)$, and ${\mathrm{dist}}_{\mathrm{dom}\_\mathrm{down}}\left(p,R\right)=\underset{R_{i}\in R}{\max}\left(\mathrm{dist}{\left(p,R_{i}\right)}_{\min}\right)=\underset{q_{i}\in Q}{\max}\left(\mathrm{dist}\left(p,q_{i}\right)-r\right)$.
3. By condition (6), $\forall p\in P-\left\{p^{*}\right\}$, satisfy ${\mathrm{dist}}_{\mathrm{dom}\_\mathrm{up}}\left(p^{*},R\right)\leq {\mathrm{dist}}_{\mathrm{dom}\_\mathrm{down}}\left(p,R\right)$.
4. $∴\underset{q_{i}\in Q}{\max}\left(\mathrm{dist}\left(p^{*},q_{i}\right)+r\right)\leq \underset{q_{i}\in Q}{\max}\left(\mathrm{dist}\left(p,q_{i}\right)-r\right).$
5. $∴r\leq \frac{\underset{q_{i}\in Q}{\max}\left(\mathrm{dist}\left(p^{*},q_{i}\right)\right)-\underset{q_{i}\in Q}{\max}\left(\mathrm{dist}\left(p,q_{i}\right)\right)}{2}.$
6. $∴\forall p\in P-\left\{p^{*}\right\}$, $r\leq \frac{\mathrm{dist}{\left(p,Q\right)}_{\max}-\mathrm{dist}{\left(p^{*},Q\right)}_{\max}}{2}$.
7. $∴\mathrm{take}\;\mathrm{the}\;\mathrm{minimum}\;\mathrm{of}\;\mathrm{dist}{\left(p,Q\right)}_{\max}$.
8. $∴r_{\max}=\frac{\underset{p\in P-\left\{p^{*}\right\}}{\min}\left(\mathrm{dist}{\left(p,Q\right)}_{\max}\right)-\mathrm{dist}{\left(p^{*},Q\right)}_{\max}}{2}.$

□

In fact, p is the second aggregate nearest neighbor $p_{\mathrm{ann}}^{2}$ of the group of users. The purpose of constructing the secure area is to reduce the frequency of communication between the user and the LSP, thereby, reducing the probability that the LBS server reconstructs the user’s trajectory, which is suitable for continuous queries.

Therefore, a circular secure areas construction algorithm is shown in Algorithm 1.

**Algorithm 1:** Circular secure areas construction **Input:**
P,Q **Output:**
R

$\mathrm{while}\;Q_{\mathrm{current}}\;\mathrm{not}\;\mathrm{in}\;\times_{i=1}^{n}R_{i}\;\mathrm{do}$
$p^{*}\leftarrow \mathrm{SOANN}\left(p,Q_{\mathrm{current}}\right)\;$                                              // Algorithm 4$p\leftarrow \mathrm{SOANN}\left(P-\left\{p^{*}\right\},Q_{\mathrm{current}}\right)$                                  // Algorithm 4${\mathrm{compute}\;r}_{\max}\leftarrow \;\frac{\underset{p\in P-\left\{p^{*}\right\}}{\min}\left(\mathrm{dist}{\left(p,Q\right)}_{\max}\right)-\mathrm{dist}{\left(p^{*},Q\right)}_{\max}}{2}$       // Formula (7)
$\mathrm{for}{\;\mathrm{each}\;q}_{i}\in Q_{\mathrm{current}}\;\mathrm{do}$

$R_{i}\leftarrow C\left(q_{I},r_{\max}\right)$

end for

end while



In Algorithm 1, the first line limits the condition to enter the loop body, which includes two cases: (1) The group of users issue the query for the first time, that is, the secure areas have not been constructed; (2) a user in the group is about to leave the secure areas, that is, the optimal aggregate nearest neighbor changes. Lines 2–3 call Algorithm 4 (see details in Section 6) to find the optimal aggregate nearest neighbor and the second aggregate nearest neighbor. Line 4 calculates the maximum radius by Formula (7). Lines 5–7 construct circular secure areas for the group of users and monitors the users’ movement in real time. Once a user leaves his secure area, it needs to reacquire the user’s new location and send a new group request to the LSP.

## 6. ANNQ Based on Strategy Optimization

In this section, we first introduced the motivation of the section. Then, we elaborated on the search and prune phase of the ANNQ. Finally, we proposed the ANNQ, based on strategy optimization.

### 6.1. Motivation

Referring to the system architecture shown in Figure 4, we could derive the response delay (rd) of a complete query service, as shown in Formula (8).

(8) rd=cd between user and TAS ×2+anonymous processing delay+cd between TAS and LSP ×2 + ANNQ delay

Among them, cd represents communication delay, which is almost negligible, the anonymous processing delay on the TAS includes anonymous protection and filtering, and the ANNQ delay includes the traversal of the POIs index tree and filtering out the optimal location point that satisfies the user. When ‘ANNQ delay≫other delay’, the ANNQ delay becomes the main reason that affects the total service response delay. Therefore, the purpose of this section is to optimize the query algorithm.

We divide the aggregate nearest neighbor query algorithm into the search phase and the prune phase, in which the search phase determines the search scope and the prune phase determines the aggregate nearest neighbor. Furthermore, we made two improvements—(1) optimization of the search strategy and further narrowing of the search scope; and (2) optimization of the prune strategy and acceleration of the prune speed.

### 6.2. Search Phase

The purpose of the search phase is to limit the search scope of POIs, and ensure that the aggregate nearest neighbor must be within this scope, so Theorem 3 is introduced, as shown below.

**Theorem** **3.** *Given a group of users $Q=\left\{q_{i}|\begin{array}{c} n\\ i=1 \end{array}\right\}$, each user’s nearest neighbor set $S_{i}=\left\{p_{i}^{l}|\begin{array}{c} m\\ l=1 \end{array}\right\},\;\forall j<k,j,k\in \left[1,m\right]$ satisfies $\mathrm{dist}\left(p_{i}^{j},q_{i}\right)\leq \mathrm{dist}\left(p_{i}^{k},q_{i}\right)$. Then, extending the nearest neighbor set of users in a group with any search strategy, as long as $S_{1}\cap^{}S_{2}\cap^{}\cdots \cap^{}S_{n}=\left\{p_{G}\right\}$, where $p_{G}$ is the first point that all users once searched, $p_{\mathrm{ann}}\in S_{1}\cup^{}S_{2}\cup^{}\cdots \cup^{}S_{n}$. The process of proof is shown below.*

**Proof.**  Use proof by contradiction.

1. Let $S_{Q}=S_{1}\cup^{}S_{2}\cup^{}\cdots \cup^{}S_{n}$. Suppose $p_{\mathrm{ann}}=p_{G}$, but $p_{\mathrm{ann}}\notin S_{Q}$.
2. $∵p_{G}\notin S_{Q}$.
3. $∴p_{G}\notin S_{i\in \left[1,n\right]}$.
4. Combined with Theorem 3, $\mathrm{dist}\left(p_{G},q_{i}\right)\geq \mathrm{dist}\left(p,q_{i}\right),p\in S_{Q}$.
5. $∵{\mathrm{dist}}_{\mathrm{agg}}\left(p_{G},Q\right)=\sum_{i\in \left[1,n\right]}\mathrm{dist}\left(p_{G},q_{i}\right)$, ${\mathrm{dist}}_{\mathrm{agg}}\left(p,Q\right)=\sum_{i\in \left[1,n\right],j\in \left[1,m\right]}\mathrm{dist}\left(p_{i}^{j},q_{i}\right)$.
6. $∴{\mathrm{dist}}_{\mathrm{agg}}\left(p_{G},Q\right)>{\mathrm{dist}}_{\mathrm{agg}}\left(p,Q\right)$. It’s conflicted with above assumption.
7. $∴p_{\mathrm{ann}}\in S_{1}\cup^{}S_{2}\cup^{}\cdots \cup^{}S_{n}.$

□

It has been indicated that, irrespective of whether the search strategy is good or bad, it can only determine the size of $S_{Q}$ and cannot change the result of aggregate nearest neighbor query. From Theorem 3, the end condition of the computing search scope $S_{Q}$ is to find a global public nearest neighbor $p_{G}$. Therefore, the goal of this section is to obtain the $p_{G}$, as soon as possible, which is related to the specific search strategy. For example, Table 2 records the nearest neighbor sets of each user in the group, when $p_{G}$ appears. In this case, $p_{G}=p_{2}$, and the search scope $S_{Q}=\left\{p_{1},p_{2},p_{3},p_{4}\right\}$.

From the vertical view of the table, we considered the extension order of the user’s nearest neighbor set. From the horizontal view of the table, we could set the conditions that p was added to the user’s nearest neighbor set.

This study argued that the distribution of the group of users affects the results of the aggregate nearest neighbor queries. When the distribution of the group of users was not uniform, that is, there was a relative aggregate subgroup, the $p_{\mathrm{ann}}$ tended to be POI, near the aggregate subgroup. The reason was that when a POI was far from the aggregate subgroup, the distance from the POI to the aggregate subgroup would increase, and the distance from the POI to a few objects would be reduced, and the total aggregate distance would still increase. Therefore, the definition of an aggregate subgroup is as follows.

**Definition** **5.** **Aggregate Subgroup.** *Given a group of users $Q=\left\{q_{i}|\begin{array}{c} n\\ i=1 \end{array}\right\}$, each user’s nearest neighbor set $S_{i}=\left\{p_{i}^{l}|\begin{array}{c} m\\ l=1 \end{array}\right\}$, $\exists p_{L}\in S_{i}$, satisfy*

(9) $$\left|\left\{S_{i}|p_{L}\in S_{i}\right\}\right|\geq \frac{\left|Q\right|}{e}$$

*then $Q=\left\{q_{i}|\left|\left\{S_{i}|p_{L}\in S_{i}\right\}\right|\geq \frac{\left|Q\right|}{e}\right\}$ constitutes an aggregate subgroup. $\left|\left\{S_{i}|p_{L}\in S_{i}\right\}\right|$ represents the size of the aggregate subgroup. $p_{L}$ is the local aggregate nearest neighbor of the aggregate subgroup, and e is a regulator used to control the size of the aggregate subgroup.*

**Figure 9:**
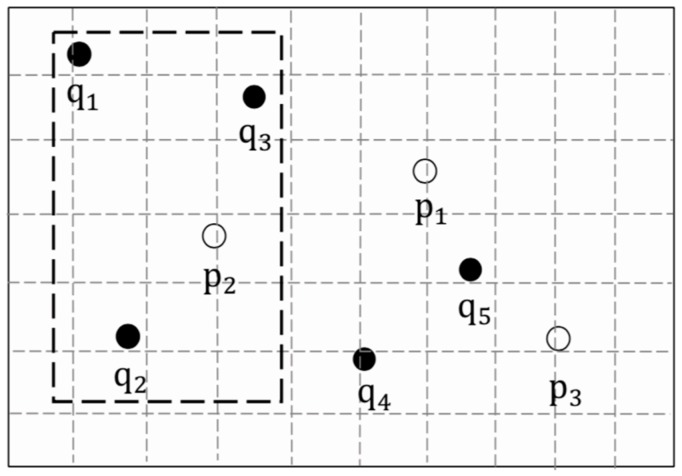
Aggregate subgroup.

As shown in Figure 9, $\left|\left\{S_{1},S_{2},S_{3}\right\}\right|=|\left\{p_{2},p_{2},p_{2}\right\}|=3>$5/2, so $q_{1}$, $q_{2}$, and $q_{3}$ constitute an aggregate subgroup, and the local public POI is $p_{L}=p_{2}$. At this point, the remaining $q_{4}$ and $q_{5}$ needed to be extended preferentially because extending the nearest neighbor set of the users outside the aggregate subgroup could search for the POIs around the aggregate subgroup, as soon as possible, thus, accelerating the appearance of the global public POI.

In addition, we also considered the direction when searching the user’s nearest neighbors. Since the gathering place of the group tended to be near their centroid, so the POIs towards the centroid should be preferentially searched, compared to the POIs in the opposite direction of the centroid.

**Definition** **6.** **Towards the Centroid.** *Given a group of users $Q=\left\{q_{i}|\begin{array}{c} n\\ i=1 \end{array}\right\}$, the centroid of group $q_{c}=\left(\frac{\sum_{i=1}^{\left|Q\right|}x_{i}}{\left|Q\right|},\frac{\sum_{i=1}^{\left|Q\right|}y_{i}}{\left|Q\right|}\right)$. Suppose the nearest neighbor set of $q_{\mathrm{cur}}$ is extended, and the current searched nearest neighbor is p. If*

(10) $$\vec{q_{\mathrm{cur}}q_{c}}\cdot \vec{q_{\mathrm{cur}}p}>0$$

*then p is in the centroid direction of $q_{c}$, else, p is in the inverse centroid direction of $q_{c}$.*

**Figure 10:**
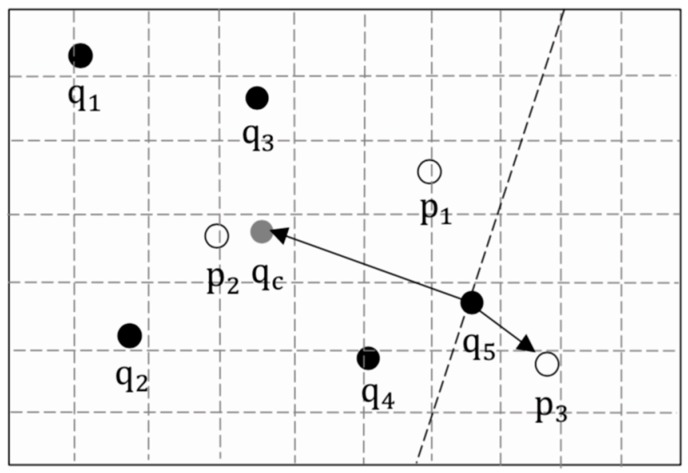
Towards the centroid.

Each user in the group follows the principle of ‘Towards the Centroid’, when extending their nearest neighbor sets, which makes it faster to find the global public POI. As shown in Figure 10, $q_{c}$ is the centroid of $q_{1}$ to $q_{5}$, $p_{3}$ is the nearest neighbor of $q_{5}$, but $\vec{q_{5}q_{c}}\cdot \vec{q_{5}p_{3}}<0$, which means that $p_{3}$ is in the inverse centroid of $q_{5}$, so $p_{3}$ would not be added to $q_{5}$’s nearest neighbor set.

Combining the above two optimizations, the pseudocode of the search phase is shown in Algorithm 2. The main idea is, first, calculate the centroid of the group, and then specify that all users search for the nearest neighbors, based on the principle of ‘Towards the Centroid’, then search for the nearest neighbor $p_{i}^{1}$ of each user, add to the nearest neighbor set $S_{i}$, and calculate $\mathrm{dist}\left(p_{i}^{1},q_{i}\right)$; second, extend the nearest neighbor set, based on the current shortest distance, until the aggregate subgroup and local public nearest neighbor are found; finally expand their nearest neighbor sets, based on the subgroup, until the global public nearest neighbor appears. The search scope is limited to the union of all users’ nearest neighbor sets.

**Algorithm 2:** Search global public nearest neighbor **Input:**
P,Q **Output:**
$S,S_{Q},p_{G},{\mathrm{dist}}_{\mathrm{agg}}$

$\mathrm{for}{\;\mathrm{each}\;q}_{i}\in Q\;\mathrm{do}$

$S_{i}\leftarrow \emptyset$

${\mathrm{search}\;q}_{i}^{\prime }{s\;\mathrm{first}\;\mathrm{NN}\;p}_{i}^{1}$

$S_{i}\leftarrow S_{i}\cup^{}\left\{p_{i}^{1}\right\},d_{i}\leftarrow \mathrm{dist}\left({\;p}_{i}^{1},q_{i}\right)$

end for

$\mathrm{compute}\;\mathrm{pd}\leftarrow \left|\left\{S_{i}{|p}_{L}\in S_{i}\right\}\right|$

$\mathrm{while}\;\mathrm{pd}<\frac{\left|Q\right|}{e}\;\mathrm{do}$

${\mathrm{select}\;q}_{\mathrm{cur}}\;\mathrm{who}\;\mathrm{has}\min\left(d_{\mathrm{cur}}\right)$

${\mathrm{search}\;q}_{\mathrm{cur}}’s\;\mathrm{next}\;\mathrm{NN}\;p\;$

$\mathrm{if}\;\vec{q_{\mathrm{cur}}q_{c}}\cdot \vec{q_{\mathrm{cur}}p}>0\;\mathrm{then}$

$S_{\mathrm{cur}}\leftarrow S_{\mathrm{cur}}\cup^{}\left\{p\right\},d_{i}\leftarrow \mathrm{dist}\left(\;p,q_{\mathrm{cur}}\right)$

$\mathrm{compute}\;\mathrm{pd}\leftarrow \left|\left\{S_{i}{|p}_{L}\in S_{i}\right\}\right|$

end while

$\mathrm{compute}\;\mathrm{Outgroup}\leftarrow \left\{q_{i}{|p}_{L}\notin S_{i}\right\}$

$\mathrm{while}\;{\cap^{}}_{i=1}^{n}S_{i}\neq \emptyset \;\mathrm{do}$

$\mathrm{for}{\;\mathrm{each}\;q}_{i}\in \mathrm{Outgroup}\;\mathrm{do}$

${\mathrm{select}\;q}_{\mathrm{cur}}\;\mathrm{who}\;\mathrm{has}\min\left(d_{\mathrm{cur}}\right)$

${\mathrm{search}\;q}_{\mathrm{cur}}’s\;\mathrm{next}\;\mathrm{NN}\;p\;$

$\mathrm{if}\;\vec{q_{\mathrm{cur}}q_{c}}\cdot \vec{q_{\mathrm{cur}}p}>0\;\mathrm{then}$

$S_{\mathrm{cur}}\leftarrow S_{\mathrm{cur}}\cup^{}\left\{p\right\},d_{i}\leftarrow \mathrm{dist}\left(\;p,q_{\mathrm{cur}}\right)$

end for

end while

$\mathrm{return}\;S,S_{Q},p_{G}={\cap^{}}_{i=1}^{n}S_{i},{\mathrm{dist}}_{\mathrm{agg}}\left(p_{G},Q\right)$



In Algorithm 2, lines 1~5 initialize the relevant variables, and lines 6~22 are the core part of the algorithm. Lines 6~13 analyzes the positional relationship of the user in the group, to determine the aggregate group. Lines 14~22 determine the search scope based on Theorem 3, ensuring that the aggregate nearest neighbor must be in it, and that the search scope is the smallest. Line 23 returns the intermediate values, such as the nearest neighbor sets and the global public POI, which is used as the input parameters for the prune phase.

### 6.3. Prune Phase

After obtaining the search scope $S_{Q}$, this section aimed to quickly prune the non-nearest neighbors. We consider a situation—when a user $q_{i}$’s nearest neighbor set has already contained all POIs in $S_{Q}$, we should not extend his nearest neighbor set any more, because if we continue to extend the user, the next nearest neighbor must not be in $S_{Q}$. Then, the prune strategy cannot prune any POI in $S_{Q}$, which is equivalent to an invalid extension.

Therefore, we should give priority to the expansion of the user whose $\left|S_{i}\right|$ is minimum because the smaller the $\left|S_{i}\right|$, the nearer the $q_{i}$ is to the aggregate nearest neighbor, the larger the prune set that can be constructed, and the more non-neighbor POIs that can be pruned. The pseudocode of the prune phase is shown in Algorithm 3.

**Algorithm 3:** Prune non-nearest neighbors **Input:**
$S,S_{Q},p_{G},{\mathrm{dist}}_{\mathrm{agg}}$ **Output:**
$p_{\mathrm{ann}}$

$\mathrm{while}\;\left|S_{Q}\right|>1\;\mathrm{do}$

${\mathrm{Search}\;q}_{\mathrm{cur}}\;\mathrm{who}\;\mathrm{has}\min\left(\left|S_{\mathrm{cur}}\right|\right)$

${\mathrm{Compute}\;q}_{\mathrm{cur}}^{\prime }s\;\mathrm{next}\;\mathrm{NN}\;p$

$S_{\mathrm{cur}}\leftarrow S_{\mathrm{cur}}\cup^{}\left\{p\right\}$

${\mathrm{dist}}_{\mathrm{agg}}^{p}\leftarrow \sum_{i=1}^{n}\mathrm{dist}\left(x_{i},q_{\mathrm{cur}}\right),x_{i}\leftarrow \{\begin{array}{c} p,\;\;p\in S_{i}\\ p_{i}^{1},\;\;p\notin S_{i} \end{array}$

$\mathrm{if}{\;\mathrm{dist}}_{\mathrm{agg}}^{p}>{\mathrm{dist}}_{\mathrm{agg}}\;\mathrm{then}$

$\mathrm{for}{\;\mathrm{each}\;q}_{i}\in Q$ **do**

$\mathrm{if}\;p\in S_{i}$ **then**

$S_{i}^{p}\leftarrow \left\{o|\mathrm{dist}\left(o,q_{i}\right)\geq \mathrm{dist}\left(p,q_{i}\right)\right\}$

else

$S_{i}^{p}\leftarrow \emptyset$

end for

$S_{Q}\leftarrow S_{Q}-\left({\cup^{}}_{i=1}^{n}S_{i}^{p}\right)$

else

$\mathrm{if}\;p\in {\cap^{}}_{i=1}^{n}S_{i}$ **then**

$S_{Q}\leftarrow S_{Q}-\left\{p\right\}$

$p_{G}\leftarrow p$

${\mathrm{dist}}_{\mathrm{agg}}\leftarrow \mathrm{dist}\left(p_{G},Q\right)$

end if

end while

$\mathrm{output}{\;p}_{\mathrm{ann}}=p_{G}$



In Algorithm 3, the first line determines whether there is any POI in the search scope $S_{Q}$. If so, it enters the loop body, else, it exits the loop. In the loop body, lines 2~5 extend the user $q_{i}$ with the smallest nearest neighbor set $\left|S_{i}\right|$, by an efficient expansion mode. Lines 6~13 construct the prune set $S_{i}^{p}$ and prune from $S_{Q}$. Lines 14~20 update the current aggregate nearest neighbor. Finally, when $\left|S_{Q}\right|=1$, the aggregate nearest neighbor is the output.

The So-ANN query algorithm in this study is composed of Algorithms 2 and 3, as shown in Algorithm 4.

**Algorithm 4** ANNQ based on strategy optimization **Input:**
P,Q **Output:**
$p_{\mathrm{ann}}$
SGPNN()                                                             //Algorithm 2PNN()                                                                  //Algorithm 3


The algorithm calculates the aggregate nearest neighbor of a group, by extending the nearest neighbor set of each user, and the most frequent operation ‘earch q’s next NN p’ is hardly time consuming. Fortunately, with the support of Spatial Oracle’s spatial query component, it can create a kNN index, based on the current location of each user, once the POIs dataset is loaded into an Oracle database. Therefore, the algorithm does not need to care about how to search the next nearest neighbor of the user, and only needs to record the subscript of the nearest neighbor that is currently added to the user’s nearest neighbor set. The time complexity of Algorithm 4 is O(|Q|) and the spatial complexity is $O\left(\left|S\right|+\left|s_{Q}\right|\right)$.

## 7. Simulation Experiment and Analysis

In this section, the experimental environment and parameter settings are introduced. Then, the state of security that the PCANNQ scheme can achieve is analyzed, and compared to the processing time ratio on the TAS and the LSP. Finally, we compared the performance of the SOANN with peer algorithms.

### 7.1. Experimental Setup

Our experiments were implemented by the Java Development Kit (JDK)-1.7 and Eclipse Integrated Development Environment (IDE), running on two local machines with an Intel Core-i7 2.5 GHz, 8 GB RAM, and Microsoft Windows 7 OS, to simulate the TAS and the LSP, in our system architecture.

As shown in Table 3, we used the simulator Network-Based Generator of Moving Objects [31], to generate mobile nodes and simulate their movement on the real map of Dongcheng District, Beijing, which was extracted from the Open Street Map (https://www.openstreetmap.org/), as well as the corresponding POIs. The district covered an area of approximately 5 km×13 km, with about 165,326 POIs, including various POI types, such as shopping, dining, medical care, and scenic spots. We stored shopping POIs and medical POIs in the Oracle Spatial and organized them with the R tree, because the distributions of these two types of POIs were significantly different, which was conducive to our experimental comparison.

We explored several factors in our experiments as summarized in Table 4, with default values shown in bold. In every set of experiments, we only changed one parameter, with the rest set as their defaults. All default parameter values have been included in this table unless otherwise noted.

It is worth mentioning that the generation of the initial location of the group of users followed the following principles: $\forall Q=\left\{q_{i}|\begin{array}{c} n\\ i=1 \end{array}\right\}$, satisfy that convex hull(Q) covers the entire map. This is consistent with the scenario in the study that required users to converge from all directions. Additionally, the speed of users could be set to fast, medium, and slow, in Brinkhoff, respectively.

The experiment consisted of two parts. In the first part, we evaluated the security of our PCANNQ scheme. We selected the CSKA [18] scheme as the baseline algorithm, compared these two schemes in terms of the abilities to resist the LSP from inferential attack and spatial-temporal correlation attack, and further analyzed the advantage of our scheme. In the second part, we evaluated the performance of our PCANNQ scheme. We selected the VANN [26] and voronoi-based range spatial skyline algorithm (VRSSA) [7] as the baseline algorithms, compared these algorithms in terms of response delay, and further analyzed the key factors that affected the efficiency. All experiments were performed a 1000 times and the average was taken as the final comparison result.

### 7.2. Security Comparison

We focused on location security in the snapshot queries and the trajectory security in the continuous queries. The location security in the snapshot queries was guaranteed by entropy, which indicated the probability that the LSP determined the real location from the set candidate. A higher entropy meant a higher location security.

**Figure 11:**
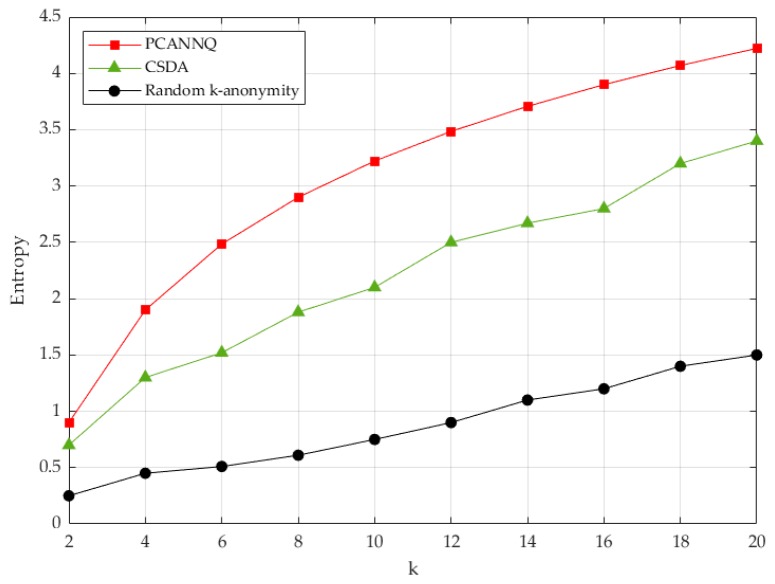
Security of locations.

As shown in Figure 11, the spatial k-anonymity method used in our PCANNQ scheme could achieve the maximum entropy under different anonymity degrees, which was superior to the random k-anonymity method and the CSDA scheme. The reason was that we selected the locations which had the same query probability with the real location as the dummy locations, to prevent the LSP from inference attack, based on the background knowledge. Although the CSDA scheme did not take into account the background knowledge of the LSP, the CSDA scheme was still better than the random k-anonymity method because it did not generate dummy locations in impossible areas, such as lakes, mountains, etc.

The trajectory security in continuous queries was guaranteed by η, which indicated the ratio of the actual number of query submissions to the total number of query submissions. Fewer the times that the queries were actually issued, the less private information was exposed to the LSP. In other words, we could improve the security of the trajectories by reducing the number of actual submitted queries.

**Figure 12:**
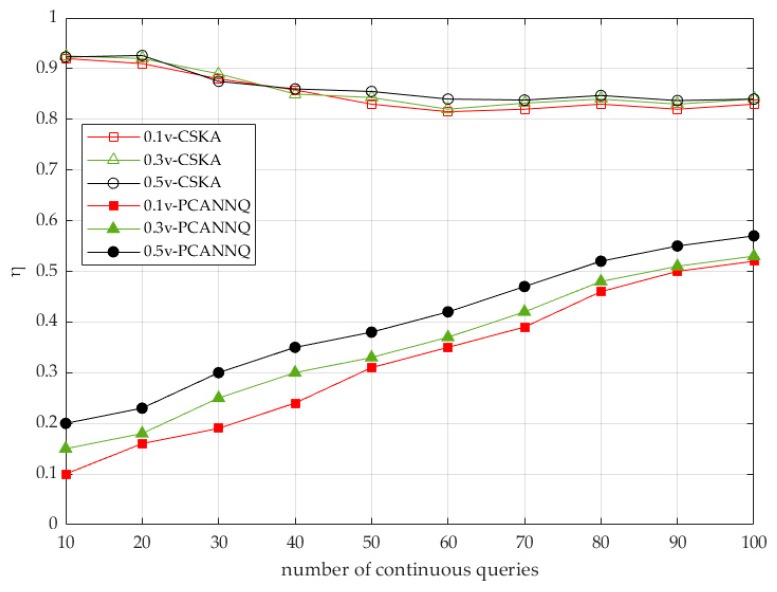
Security of trajectories.

Figure 12 shows the users’ query submissions in continuous queries. In the CSKA scheme, (1) as the number of continuous queries increased, η gradually decreased and tended to be stable, since at the beginning, the hit rate was low, due to the small contents in the cache, but this situation was alleviated as the number of queries increased. (2) This method could reduce the submission of query requests, but η was always large in general (>0.8), because the query object was a group of users, not a single user, so the hit rate of similar queries in the TAS’s cache was very low. (3) The mobile speed of users had almost no impact on η, because the caching technology had nothing to do with user speed.

In our PCANNQ scheme, (1) as the number of continuous queries increased, η increased. The reason was that, the more continuous the queries, the greater the moving distance of users, which led to a more frequent replacement of the optimal aggregate nearest neighbor. (2) Although the number of continuous queries had increased, η could still maintain a low value (<0.6) because the secure areas concealed the submission of partial query requests. (3) The user’s moving speed affected η, and the faster the speed, the larger η. The reason was that, the faster the user moved, the more times the secure areas were exceeded, which led to more query requests that needed to be submitted.

To sum up, the advantage of our PCANNQ scheme was that, we actively constructed secure areas to conceal part of the users’ trajectories, which not only overcame the problem of cold starts in the cache, but also greatly reduced the number of actual submitted queries. Therefore, our PCANNQ scheme could achieve a higher trajectory security. Then, we further analyzed the factors that affected the secure areas.

**Figure 13:**
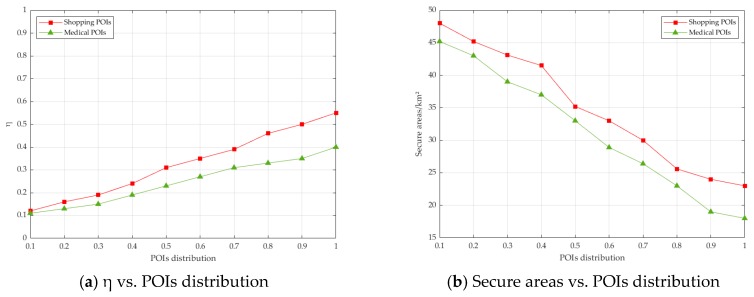
The effect of points of interest (POIs) distribution on η and Secure areas.

Figure 13a analyzes the effect of the distribution of POIs on η. The experimental results showed that the sparser the distribution of POIs, the larger was the η. Combined with Section 5, we knew that the calculation of the radius of secure areas depended on the calculation of $\mathrm{dist}{\left(p_{\mathrm{ann}}^{1},Q\right)}_{\max}$ and $\mathrm{dist}{\left(p_{\mathrm{ann}}^{2},Q\right)}_{\max}$. The sparser the distribution of POIs, the larger was the radius and the area of the secure areas are, so η was smaller. Figure 13b also verifies that η was negatively correlated with the area of the secure area. Additionally, the shopping POIs itself were denser than the medical POIs, in terms of the distribution characteristics. The experimental data showed that shopping POIs needed more data submissions than the medical POIs. Therefore, there was a suggestion that if the POIs information of the query was sensitive to the user, we could increase the distribution of such POIs, so that the correlation between the trajectory and the POIs information could be reduced.

Finally, the experiment explored the applicable scope of our PCANNQ scheme. We set the number of users for n as 5, and the continuous query passed 1000 timestamps. In theory, the higher the moving speed, the higher the communication frequency, because the faster the speed, the faster the secure area will be exceeded.

**Figure 14:**
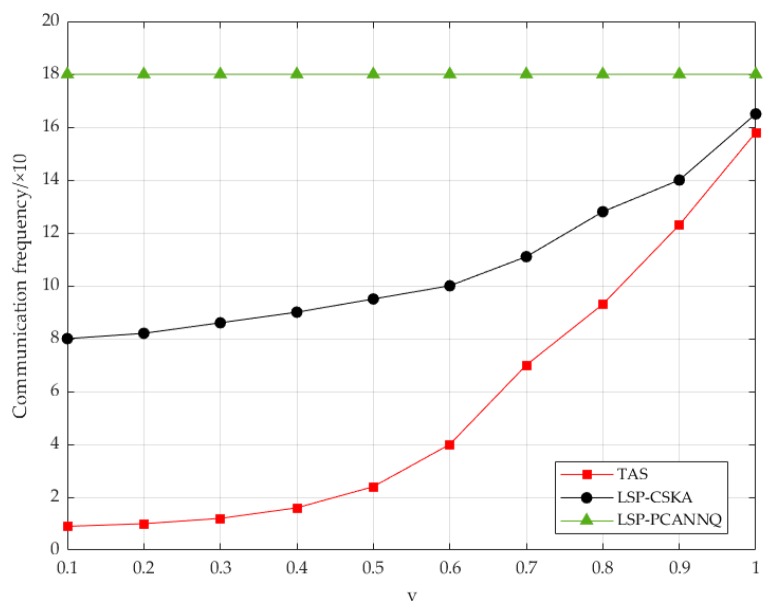
The effect of moving speed on communication frequency.

As shown in Figure 14, as the speed increased, the communication frequency between the user and the LSP in our PCANNQ scheme showed an exponentially increasing trend. The user communicated with the TAS in real-time, so the communication frequency between the user and the TAS was constant. The difference between the two lines in the figure was the number of communications concealed by secure areas. Additionally, from 0.1 v to 0.6 v, a lower communication frequency could be guaranteed, and the speed of this level was equivalent to the speed of human walking and even the speed of a human riding. When the moving speed was greater than 0.6 v, the gap between our PCANNQ scheme and the CSKA scheme gradually decreased. Therefore, this algorithm was more applicable to the continuous query under the speed range between 0.1 v and 0.6 v.

### 7.3. Efficiency Comparison

**Figure 15:**
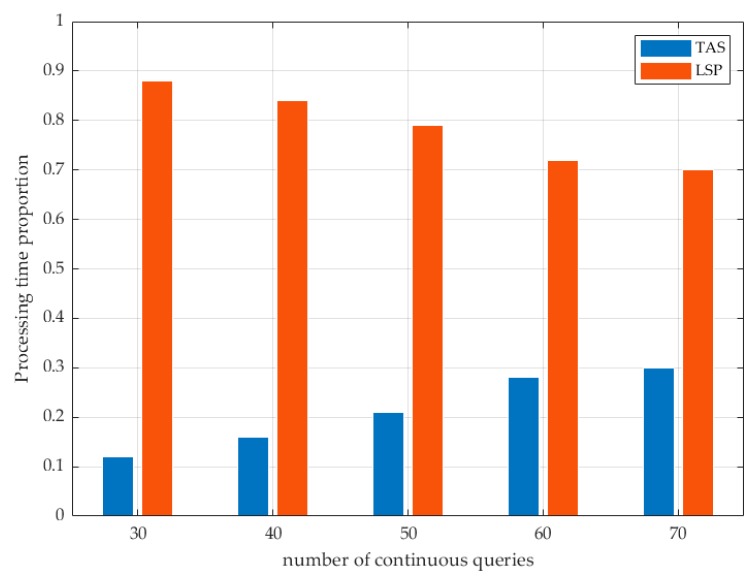
Service processing time proportion.

As show in Figure 15, we calculated the proportion of the processing time of the TAS and the LSP in the total query service. (1) The processing time of the TAS was much smaller than the processing time of the LSP, which was dominant in the total processing time. (2) With the increase of the number of continuous queries, the processing overhead of the LSP had a tendency to decline. Due to the secure areas, the users did not need to continuously send all requests to the LSP and some queries returned directly from the TAS, so the processing time of the TAS had increased slightly.

Therefore, we continued to optimize the query algorithm. The following experiment verified the performance of the SOANNQ proposed in this paper. We mainly explored the relationship between the query efficiency and the number of users and the degree of distribution between users.

**Figure 16:**
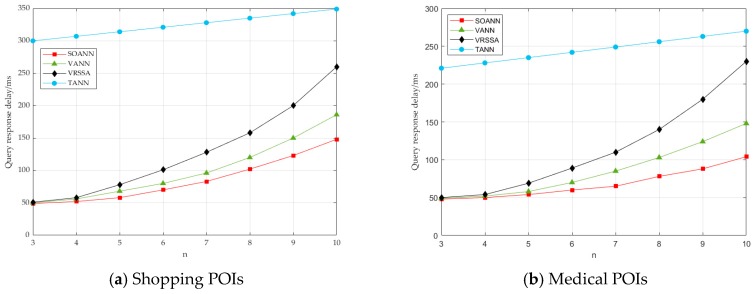
The effect of number of users on query response time.

In Figure 16a,b, the user distribution was first made constant (a = 3%), and then the shopping POIs and the medical POIs were searched separately. The following points could be drawn from the figure. (1) The SOANN algorithm showed obvious advantages in the query response relay, which proved that this algorithm improved the VANN algorithm and had advantages over the VRSSA algorithm. (2) The SOANN algorithm showed linear growth, the VANN and VRSSA algorithms showed exponential growth. The larger was the n, the longer was the query response delay. (3) The SOANN and VANN algorithms were not sensitive to the type and distribution of POI. However, the TANN algorithm and the VRSSA algorithm were more sensitive to the type and distribution of POI, because the former did not need to retrieve all POI information, but the latter did need them.

**Figure 17:**
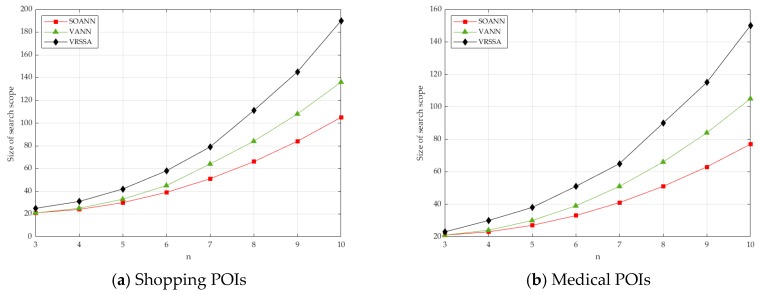
The effect of number of users on the searching set size.

In Figure 17a,b, the reason why the SOANN algorithm was superior to the VANN algorithm and the VRSSA algorithm is shown. This was because the key factor that influenced the query response time was whether the search scope could be minimized. It can be seen from the figure that the search scope constructed by the SOANN algorithm was the smallest, and with the increase of n, the change trend was the same, with a response delay of the query, which also proved the effectiveness of the searching strategy optimization proposed in this study.

Finally, we explored the influence of user distribution on the query response time. The user distribution was the ratio of the number of POIs and the total number of POIs included in the convex hull formed by the users in the group. The number of users in the group was n = 5.

**Figure 18:**
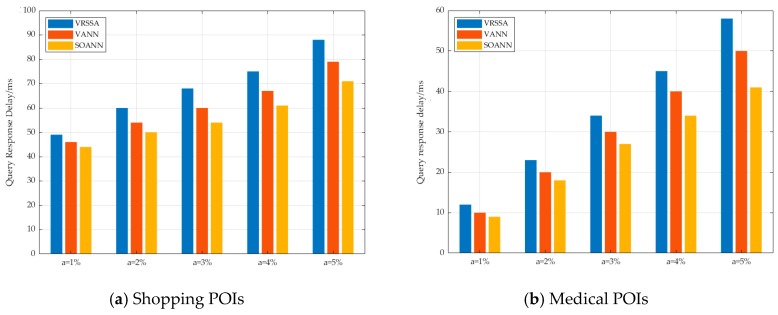
The effect of user distribution on query performance.

Figure 18 shows that when the user distribution in the group was sparse, that is, the value of ‘a’ was large, the SOANN algorithm performed better because the SOANN algorithm analyzed the distribution of users in the group and preferentially extended the outlier users. The VANN algorithm extended all users, one by one, in accordance with the principle of fairness, so the constructed search scope was also larger and the query response time was longer. The VRSSA algorithm needed to calculate all POIs contained in a convex hull, composed of the group of users, consequently, the cost became very expensive.

In summary, it could be concluded that our SOANN algorithm showed a good efficiency when the number of users was large and the distribution was dispersed.

## 8. Conclusions

In order to deal with the leakage of location and trajectory privacy faced by the IOT, when providing query services, we proposed a secure and efficient PCANNQ scheme to protect the location and trajectory privacy of the group of users. Specifically, the entropy based spatial K-anonymity method was used to prevent the LSP from inference attacks and protect the location privacy of the group of users. The circular secure areas were used to deconstruct the continuous queries into uncorrelated snapshot queries, and conceal part of the users’ trajectories to prevent the LSP from spatial-temporal correlation attacks. Then, the aggregate nearest neighbor query algorithm, based on strategy optimization, was used to further reduce the overall delay of the service. Finally, the simulation results also verified that our proposed scheme could ensure a high privacy security, and that the response speed and communication overhead of the service were superior to other peer algorithms, both, in the snapshot and the continuous queries. In addition, although this article considered the user’s mobility, it did not consider that often the user would be moving along the road network. The next step could be considered to extend the Euclidean space to the road network space.

## Figures and Tables

**Table 1:** Summary of notations.

Notation	Description	Notation	Description
P	set of POIs	$p_{G}$	global public nearest neighbor
Q	set of users	$p_{L}$	local public nearest neighbor
$S_{q}$	nearest neighbor set of user q	$S_{\mathrm{prun}}$	prune set
$S_{Q}$	nearest neighbor set of group Q	$p_{\mathrm{ann}}^{k}$	the kth aggregate nearest neighbor
dist(p,q)	distance between p and q	$p^{*}$	dominated POI
${\mathrm{dist}}_{\mathrm{agg}}\left(p,Q\right)$	aggregate distance between p and Q	R	set of secure areas
$p_{i}^{j}$	the jth nearest neighbor of $q_{i}$	$R^{*}$	set of maximum secure areas

**Table 2:** Example of the nearest neighbor sets at the end of the search phase.

Q	S	D/km
$u_{1}$	$S_{1}=\left\{p_{1},p_{3},p_{2}\right\}$	$D_{1}=\left\{1.1,2.1,3.5\right\}$
$u_{2}$	$S_{2}=\left\{p_{2},p_{4}\right\}$	$D_{2}=\left\{1.0,2.3\right\}$
$u_{3}$	$S_{3}=\left\{p_{2}\right\}$	$D_{3}=\left\{3.0\right\}$

**Table 3:** Data sets.

Type	Name	Amount
POI set	Dongcheng District, Beijing	165,326
Trajectory set	Thomas Brinkhoff	120

**Table 4:** Parameters and their values.

Description	Notation	Ranges	Default Values
Number of users	n	3–10	5
Number of POIs	m	5 K–15 K	10 K
Distribution of users	a	1–5%	3%
Moving speed	v	5–100 km/h	50 km/h
Distribution of POIs	d	10–100%	50%
Anonymity degree	k	2–10	6
Number of continuous queries	q	10–100	50
